# Targeted therapy for pancreatic ductal adenocarcinoma: Mechanisms and clinical study

**DOI:** 10.1002/mco2.216

**Published:** 2023-02-19

**Authors:** Heng‐Chung Kung, Jun Yu

**Affiliations:** ^1^ Krieger School of Arts and Sciences Johns Hopkins University Baltimore Maryland USA; ^2^ Departments of Medicine and Oncology Johns Hopkins University School of Medicine Baltimore Maryland USA

**Keywords:** neoadjuvant therapy, pancreatic ductal adenocarcinoma (PDAC), randomized clinical trials, signaling pathways, targeted therapy

## Abstract

Pancreatic ductal adenocarcinoma (PDAC) is an aggressive and lethal malignancy with a high rate of recurrence and a dismal 5‐year survival rate. Contributing to the poor prognosis of PDAC is the lack of early detection, a complex network of signaling pathways and molecular mechanisms, a dense and desmoplastic stroma, and an immunosuppressive tumor microenvironment. A recent shift toward a neoadjuvant approach to treating PDAC has been sparked by the numerous benefits neoadjuvant therapy (NAT) has to offer compared with upfront surgery. However, certain aspects of NAT against PDAC, including the optimal regimen, the use of radiotherapy, and the selection of patients that would benefit from NAT, have yet to be fully elucidated. This review describes the major signaling pathways and molecular mechanisms involved in PDAC initiation and progression in addition to the immunosuppressive tumor microenvironment of PDAC. We then review current guidelines, ongoing research, and future research directions on the use of NAT based on randomized clinical trials and other studies. Finally, the current use of and research regarding targeted therapy for PDAC are examined. This review bridges the molecular understanding of PDAC with its clinical significance, development of novel therapies, and shifting directions in treatment paradigm.

## Graphical abstract

Pancreatic ductal adenocarcinoma (PDAC) is an aggressive and lethal malignancy with a high rate of recurrence and a dismal 5‐year survival rate. A recent shift toward a neoadjuvant approach to treating PDAC has been sparked by the numerous benefits neoadjuvant therapy (NAT) has to offer compared with upfront surgery. This review bridges the molecular understanding of PDAC with its clinical significance, development of novel therapies, and shifting directions in treatment paradigm with an emphasis on the use of NAT.

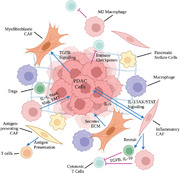

## 1 INTRODUCTION

Pancreatic ductal adenocarcinoma (PDAC) was the fourth leading cause of cancer‐related death in 2021 and is projected to be second by 2030.^1^, ^2^ It is associated with a poor 5‐year survival rate of 11%, most likely due to its tendency to disseminate early‐on, lack of effective systemic therapy, and delayed response.^3^, ^4^, ^5^ Surgical resection is the only cure for PDAC currently, but patients often present with metastatic disease or unresectable primary tumors at diagnosis.^6^ Furthermore, surgical resection alone is insufficient: up to 80% of patients with localized tumors who undergo surgical resection with curative intent experience recurrence.^6^

Over the past few decades, randomized clinical trials (RCTs), including the Gastrointestinal Tumor Study Group's (GITSG), European Study Group for Pancreatic Cancer 1 (ESPAC‐1), and the phase III CONKO‐001 trial, have demonstrated adjuvant chemotherapy's benefit on overall survival (OS) compared with resection only for patients with R0‐ and R1‐resected tumors.^7^, ^8^, ^9^ As such, adjuvant chemotherapy is currently the standard of care after surgical resection.^10^ More recently, the PRODIGE‐24/CCTG PA.6 trial showed FOLFIRINOX had better OS (53.5 vs. 35.5 months), disease‐free survival (DFS) rate (26.1 vs. 19.0%), and 5‐year OS rate (43.2 vs. 31.4%) compared with gemcitabine albeit at the expense of more toxic effects.^11^, ^12^ Consequently, FOLFIRINOX is now considered the standard adjuvant chemotherapy treatment for patients with better performance status, while gemcitabine is still recommended for people with worse performance status.

However, there has been a recent shift of focus toward a neoadjuvant approach against PDAC. Hypothesized benefits of neoadjuvant therapy (NAT) include early treatment of micrometastases, guaranteed delivery of systemic therapy, downstaging, improved rates of negative lymph nodes, enhanced ability to select patients suitable for surgery, and increased rates of margin negative (R0) resections.^6^, ^13^, ^14^ NAT is currently recommended by the National Comprehensive Cancer Network (NCCN) for patients with borderline resectable pancreatic cancer (BRPC) and locally advanced pancreatic cancer (LAPC).^15^, ^16^

Despite these possible benefits of NAT, there is no clear guide for medical oncologists in terms of deciding whether to administer NAT or not, what type of patients would benefit from NAT, what regimen should be used, and how to assess tumor response to NAT.^17^ In this review, we will summarize current research of NAT against PDAC to attempt to create a more holistic understanding and discuss current and future research directions. First, we will provide an introduction of the current understanding on the molecular mechanisms, signaling pathways, and treatment options for pancreatic cancer. Then, we will address the ongoing debate on the use of NAT and the basic rationale for and against it. Next, we will discuss whether NAT should be administered based on different factors. First, the use of NAT is assessed based on the resectability of the tumor with an emphasis on evidence from RCTs. Additionally, we will highlight recent developments in the use of biomarkers from biopsies and resected tumors that could guide the treatment plan. Next, we will cover the different methods of monitoring response to NAT, including imaging, biomarkers, and novel histopathological analysis. We will then focus on the retrospective studies and completed and ongoing RCTs regarding the optimal NAT regimen. Finally, we will review the current use of targeted treatment in PDAC and research direction in that field.

## 2 CURRENT UNDERSTANDING OF PDAC

### 2.1 Pathogenesis of PDAC

Despite the aggressive nature of PDAC, it is preceded by the development of noninvasive precancerous lesions years prior to the appearance of malignant cells.^18^ Several categories of precancerous lesions exist based on their size and involvement with the pancreatic ductal system.^19^ The most common premalignant lesion of PDAC is pancreatic intraepithelial neoplasia (PanIN), which are neoplasms of the pancreatic duct that are less than 5 mm.^18^, ^19^, ^20^, ^21^ Next, less than 10% of PDACs arise from intraductal papillary mucinous neoplasms (IPMNs), which are finger‐like, microcystic lesions that protrude into the pancreatic duct.^18^ Finally, mucinous cystic neoplasms are the least common precancerous lesions.^19^ They are characterized by distinctive ovarian‐type stroma that do not involve the pancreatic ductal system.^18^, ^19^ While the development and progression of precancerous lesions have been well studied, the origins of these lesions remain a controversy: studies have reported these lesions originating from both ductal and acinar cells.^22^

Classification of the precancerous lesions hold clinical significance as well. PanINs are much smaller compared with IPMNs, making them less likely to be detected incidentally.^18^, ^23^ Precancerous lesions are also categorized as low‐grade versus high‐grade morphologically: low‐grade lesions are characterized by basally oriented nuclei with mild to moderate cytological atypia, while high‐grade lesions are marked by loss of nuclear polarity, significant architectural alterations and cytological atypia.^24^ Low‐grade lesions are more commonly found compared with high‐grade lesions, but high‐grade lesions have been associated with higher risk of progression to carcinoma, and studies have shown the possible direct link between high‐grade IPMN and PDAC.^18^

### 2.2 Genetic and molecular alterations in PDAC

PDAC is a heterogeneous disease marked by four common genetic alterations: *KRAS* oncogenic activation and *CDKN2A*, *TP53, SMAD4* tumor suppressor gene inactivation.^25^ Activating *KRAS* mutations are the earliest and most common genetic alterations to occur in PDAC progression; they are present in greater than 90% of low‐grade PanINs and 90–95% of PDAC.^26^, ^27^, ^28^ *KRAS* encodes a membrane‐bound GTPase that regulates a range of critical intracellular processes such as cell growth, differentiation, and survival by moving between its GDP‐bound inactive and GTP‐bound active form.^25^, ^29^ KRAS is usually present in quiescent cells in its inactive form; upon growth stimulation, guanine nucleotide exchange factors (GEFs) catalyze the GDP–GTP exchange to change KRAS into its active state.^30^, ^31^ Most activating *KRAS* mutations result in impairment of the protein's GTPase, causing KRAS to be locked in its GTP‐bound active form and continued activation of major downstream pathways.^32^

While activating *KRAS* mutations are often one of the earliest mutations and promote PDAC initiation, research has suggested that *KRAS* mutations alone are incapable of promoting PDAC progression.^25^, ^33^ Located on chromosome 9, *CDKN2A* encodes the p16^INK4A^ protein, which negatively regulates cell cycle progression from G1‐phase to S‐phase by disrupting complex formation between CDK4/6 and cyclin D.^34^, ^35^ Loss of *CDKN2A* is key in the progression of PDAC as it allows cells with activating *KRAS* mutations to escape cell senescence.^36^ Another one of the four commonly mutated driver genes, *TP53* encodes transcription factor p53, which binds onto DNA to promote transcription of genes (e.g., *CDKN1A*, *BAX*, etc.) that trigger cell cycle arrest or apoptosis upon DNA damage.^37^, ^38^ *TP53* mutations that result in functional loss of the transcriptional factor are observed in 50–75% of PDAC patients and typically occur following loss of *CDKN2A*.^39^

Finally, SMAD4 is a crucial mediator in the transforming growth factor‐ß (TGF‐ß) family signal transduction, which has been found to have context‐dependent and often contradictory roles in PDAC pathogenesis.^40^ In nonmalignant cells, TGF‐ß signaling directly promotes tumor suppression by inhibiting expression of cyclin‐dependent kinases (CDKs) and enhancing expression of CDK inhibitors (e.g., p15 and p21).^41^ On the other hand, TGF‐ß signaling can act as a tumor promoter in malignant cells by inducing epithelial‐to‐mesenchymal transition (EMT) and creating an immunosuppressive environment.^41^ Loss of SMAD4 function inhibits the antiproliferative effect of TGF‐ß and enhances tumor aggressiveness.^42^

In addition to these four driver genes, a wide range of genes are also mutated in different subsets of PDAC, including *BRCA1*/2, *PALB2*, *ATM*, *ARID1A, KMT2D, BRAF, FGFR1*, albeit at a lower frequency (≤10%).^43^, ^44^, ^45^, ^46^, ^47^ These alterations converge to disrupt several core cellular signaling pathways and processes, including KRAS signaling, TGF‐ß signaling, DNA damage control, apoptosis, regulation of G1/S phase transition, and Hedgehog signaling.^25^, ^48^

### 2.3 Signaling pathways in PDAC tumorigenesis and metastasis

A number of signaling pathways have been identified to play critical roles in the tumorigenesis, prognosis, metastasis, and response to treatment of PDAC, including RAS and its downstream pathways, JAK/STAT pathway, Wnt/β‐Catenin pathway, and Notch pathway.^49^, ^50^

#### 2.3.1 NF‐κB

Given that KRAS, a specific protein isoform of RAS, is the one of the earliest and most commonly mutated genes in PDAC, the RAS pathway plays a major role in PDAC initiation.^51^, ^52^ Activated RAS drives the activation of downstream effector pathways such as the NF‐κB and phosphoinositide 3‐kinase (PI3K)/protein kinase B (AKT) pathways.^53^, ^54^, ^55^, ^56^ NF‐κB is a family of transcription factors that is primarily responsible for the triggering the inflammatory response in pancreatic cancer by upregulating the transcription of cytokines, chemokines, and other proinflammatory genes.^57^ For example, activated NF‐κB enters the nucleus and promotes the transcription of IL‐6, IL‐8, and IL‐18, which further activates NF‐κB in a positive feedback loop.^58^ Constitutive activation of NF‐κB can be found in ∼70% of PDAC samples, and in addition to causing an inflammatory response, NF‐κB activation has been found to be involved in PDAC tumorigenesis.^59^ Maniati et al.^60^ found that the NF‐κB synergizes with Notch pathway to inhibit expression of anti‐inflammatory nuclear receptor Pparγ and promote PDAC progression in a *KRAS^G12D^Pdx1‐cre* mouse model. Recent studies have also demonstrated NF‐κB's role in antitumor immunity: Garg et al.^61^ demonstrated that NF‐κB in pancreatic stellate cells reduces the infiltration of cytotoxic T cells into the tumor and cancer cell killing by upregulating the expression of C‐X‐C motif chemokine ligand 12 (CXCL12).

#### 2.3.2 PI3K/AKT

The PI3K/AKT pathway is also activated in PDAC through the phosphorylation of PI3K in response to oncogenic RAS.^62^ Overactivation of AKT has been observed in 30–40% of pancreatic cancer and is associated with a poorer prognosis.^63^ Upon activation of PI3K, AKT is recruited to the plasma membrane; 3‐phosphoinositide‐dependent protein kinase‐1 phosphorylates AKT at Thr308, activating AKT.^54^ Once activated, AKT can regulate a wide range of pathways, including cell proliferation, survival, migration, and invasion.^64^ One of the main downstream molecules of the PI3K/AKT pathway is mammalian target of rapamycin (mTOR), a serine/threonine kinase involved in proliferation, apoptosis, and autophagy.^65^ mTOR can be found as two complexes: mTORC1 and mTORC2. mTORC1 is sensitive to rapamycin and can be directly activated by AKT via phosphorylation of pRAS40; mTORC2 is insensitive to rapamycin but can still activated via increased AKT phosphorylation.^55^ Studies have demonstrated that the mTOR pathway is activated in pancreatic cancer cell lines and in human PDAC samples.^66^ Given the importance of the PI3K/AKT pathway in PDAC initiation and progression, several PI3K inhibitors have been developed and are currently being tested in preclinical and clinical trials as possible therapies for PDAC.^64^

#### 2.3.3 JAK/STAT

The JAK/STAT pathway has been identified to play critical roles in tumor progression in multiple cancers.^67^, ^68^ High JAK expression and activation of the IL‐6R/JAK/STAT pathway has been associated with a worse prognosis for resectable PDAC.^69^, ^70^ The JAK/STAT pathway is particularly known to modulate the immune response in the tumor microenvironment (TME).^67^, ^71^ Lu et al.^72^ reported that JAK/STAT‐mediated chronic inflammation impairs cytotoxic T cell activation and reduces the efficacy of anti‐PD1 treatment in pancreatic cancer. Biffi et al.^73^ identified that tumor‐derived IL‐1 upregulated expression of LIF and induced downstream JAK/STAT signaling to favor the development of inflammatory cancer‐associated fibroblasts (iCAFs), whereas this process is antagonized by TGFß signaling to favor the development of myofibroblastic cancer‐associated fibroblasts (myCAFs). iCAFs are located distant from tumor cells but are known to recruit and regulate immunosuppressive cells by secreting factors such as IL‐6, CXCL2, CXCL12, and CXCL8.^74^, ^75^ Furthermore, in an in vitro study, Doi et al.^76^ reported that PD‐L1 surface expression was upregulated in pancreatic cancer cell lines AsPC‐1, MIA PaCa‐2, and Pan02 after stimulation with 5‐fluorouracil, gemcitabine, or paclitaxel. They found a corresponding increase in phosphorylation of STAT1 and total STAT1 in AsPC‐1 cells after chemotherapy treatment and a dose‐dependent reduction in PD‐L1 upregulation when treated with a JAK2 inhibitor, demonstrating the JAK/STAT pathway's involvement in PD‐L1 upregulation.^76^

#### 2.3.4 Wnt signaling

Wnt signaling is involved in multiple processes, including normal embryogenesis, organogenesis, and homeostasis. It can be further categorized into canonical and noncanonical pathways.^77^, ^78^ Under normal physiological conditions, cytoplasmic β‐catenin is targeted for degradation through phosphorylation and ubiquitination in the absence of Wnt signaling ligands.^79^ The canonical Wnt pathway is activated by Wnt family ligands binding to Frizzled/low‐density lipoprotein receptor complexes, triggering a cascade of events that eventually leads to β‐catenin stabilization, accumulation, and nuclear translocation.^80^, ^81^ β‐Catenin binds to transcription factors of the Tcf/Lef family to form a complex that upregulates the transcription of genes involved in tumor‐promoting processes, including cell cycle progression, angiogenesis, and EMT.^82^, ^83^ The Wnt pathway can also be activated by noncanonical Wnt ligands, such as GATA6, R‐spondin, MUC1, and MUC4, resulting in pancreatic cancer progression.^82^, ^84^ By inactivating β‐catenin in pancreatic epithelial cells of a KRAS‐driven mouse model of pancreatic cancer (KC), Zhang et al.^85^ showed that ligand‐mediated activation of the Wnt/β‐Catenin pathway is necessary for the initiation and progression of PDAC. Similarly, Morris et al.^86^ demonstrated that Wnt signaling is required for the development of KRAS‐induced PanIN lesions and pancreatic cancer in genetically engineered mice. Other researchers have demonstrated that epithelial Wnt signaling is involved in PDAC carcinogenesis, progression, metastasis, and chemoresistance.^87^, ^88^, ^89^, ^90^, ^91^ More recently, Wnt components were also discovered in tumor infiltrating lymphocytes (TILs) through single‐cell analysis of human pancreatic cancer.^92^ Particularly, CD4+ TILs were found to express TCF7, which encodes TCF1, a transcription factor and mediator for Wnt signaling.^92^ Conditional inactivation of *Tcf7* in CD4+ T cells in a pancreatic cancer mouse model resulted in changes in the immune TME, such as more CD8+ T cells, less T‐regulatory cells, and compensatory upregulation of PD‐L1.^92^

### 2.4 EMT in PDAC

EMT is a dynamic and biologically critical process that plays a role in mediating PDAC's highly aggressive, immunosuppressive, and resistant nature.^93^, ^94^ In different tissues of the body, epithelial cadherin molecules known as cell surface E‐cadherin help epithelial sheets maintain their structural integrity.^95^, ^96^ During EMT, cancer cells lose their epithelial features and markers, including E‐cadherin, occludin, claudin, and begin expressing mesenchymal markers such as N‐cadherin, fibronectin, and vimentin.^97^, ^98^, ^99^ Morphologically, cells undergoing EMT change from polygonal shapes to spindle‐shaped while also leading to the disruption of normal cell–cell and cell–matrix adhesion, remodeling of cytoskeleton, and loss of cellular polarity.^100^ Once cancer cells undergo EMT, they acquire migratory and invasive capabilities that help facilitate growth and metastasis while becoming more resistant to chemotherapy and other cytotoxic therapies.^101^, ^102^, ^103^, ^104^ EMT is modulated by a complex network of epigenetic modifications, transcription regulation, and multiple signaling pathways regulated by major EMT‐inducing transcription factors (EMT‐TFs) Snai1 (Snail), Snai2 (Slug), Zeb1, Zeb2, and Twist (Twist basic helix‐hoop‐helix transcription factor 1).^105^, ^106^, ^107^, ^108^

A major contributor to EMT is cancer‐associated fibroblasts (CAFs).^109^ CAFs secrete a myriad of cytokines that promote EMT of PDAC cells, including TGFβ, IL‐1, and IL‐6.^110^, ^111^ Under normal TGFβ and SMAD signaling, the pathway is growth suppressive: binding of TGFβ with TGFβ receptor I/II leads to phosphorylation of SMAD2 and SMAD3, which heterotrimerize with SMAD4 and translocate to the nucleus to act as a transcription factor that downregulates c‐Myc and upregulates CDK inhibitors p15 and p27, ultimately resulting in cell cycle arrest.^112^, ^113^, ^114^ However, TGFβ signaling induces the engagement of EMT in addition to driving fibrosis and immunosuppression in the pancreatic cancer TME.^115^, ^116^ Activated SMAD2/3/4 promotes transcription of multiple aforementioned EMT‐TFs.^117^, ^118^, ^119^ Furthermore, IL‐1α/β mediates a sustained inflammatory TME, which is critical driving mechanism of EMT, through the NF‐κB pathway.^120^, ^121^, ^122^, ^123^ Expression of TNF‐α or constitutively active IKK2 are also capable of inducing a EMT phenotype by upregulating the expression of mesenchymal marker vimentin and EMT transcription factor ZEB1 while downregulating epithelial marker E‐cadherin.^124^

Within the TME, the extracellular matrix (ECM), which is primarily produced by CAFs, also contributes to promoting EMT in addition to hindering the infiltration of treatment and cytotoxic T cells.^125^ Collagen is the major component of the ECM, accounting for over 80% of ECM mass in PDAC.^126^ Ligation of ECM proteins and integrins causes clustering of integrins and the formation of focal adhesions on the cell membrane where the integrins connect to actin cytoskeleton via linker proteins.^127^, ^128^ This interaction triggers the activation of EMT‐promoting pathways, including the FAK/SFK, Rac/Cdc42, and Fyn/YES pathways.^129^ Particularly, the FAK pathway can promote EMT by upregulating N‐cadherin expression through the JNK signaling pathway.^130^, ^131^, ^132^ FAK can also activate Yes‐associated protein (YAP) and Transcriptional Coactivator with PDZ‐Binding Motif (TAZ), forming the YAP–TAZ complex that translocates to the nucleus to transactivate genes that code for EMT‐TFs.^133^, ^134^ The dense ECM also limits vascularization and perfusion, creating a hypoxic TME that heightens chemoresistance and tumor aggressiveness.^135^, ^136^, ^137^ Hypoxia‐inducible factors (HIFs) are activated to help cells adapt to the hypoxic environment through transcriptional regulation; of note, HIF‐1α also binds to the promoter region of EMT‐TFs.^138^, ^139^, ^140^ Despite the heavy emphasis on CAFs role in promoting EMT, other cells such as tumor‐associated macrophages (TAMs) play a role in inducing a mesenchymal phenotype in PDAC cells.

### 2.5 TME of PDAC

**FIGURE 1:**
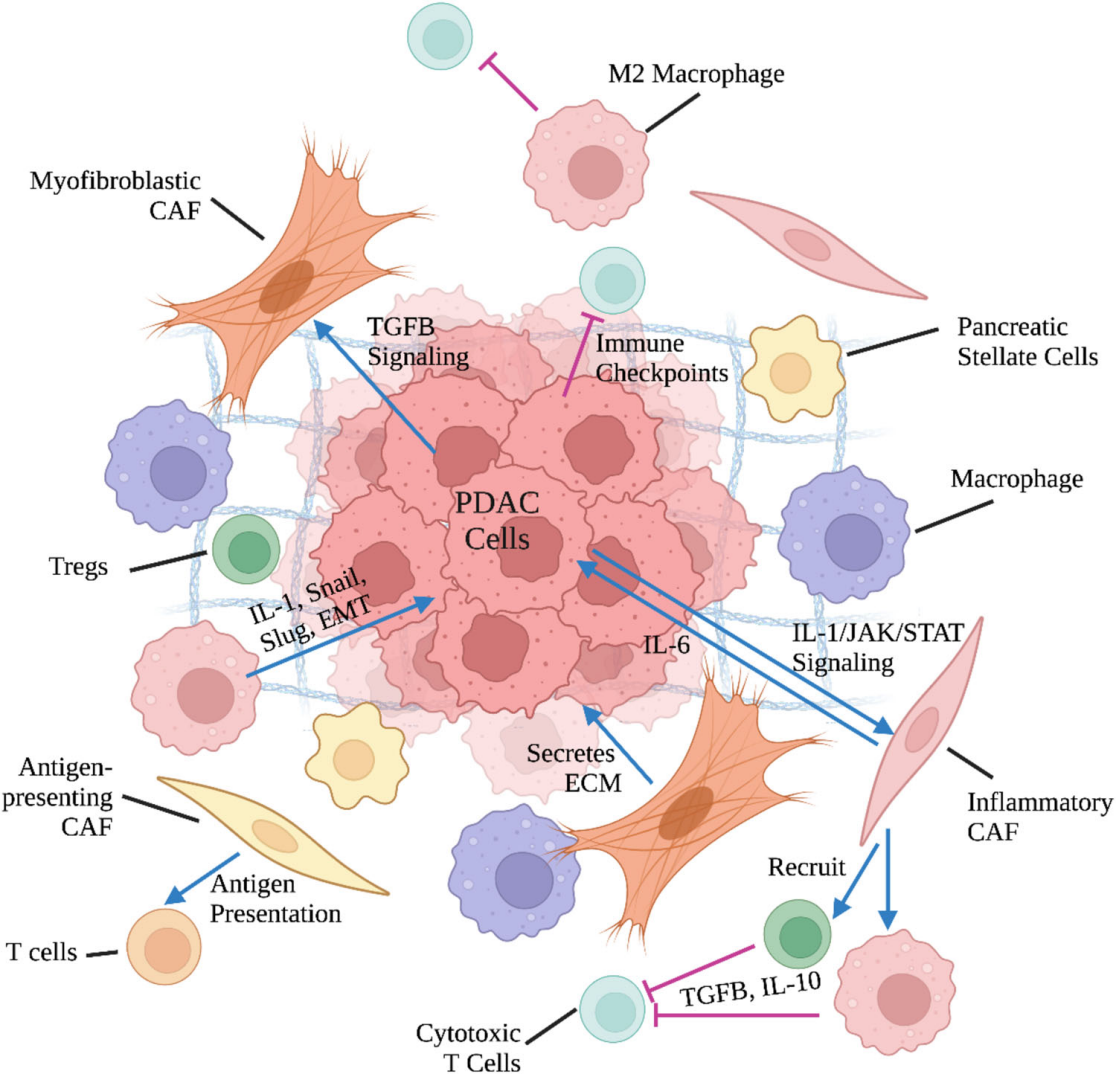
Tumor microenvironment of PDAC. The tumor microenvironment of PDAC is extremely complex. Cancer‐associated fibroblasts (CAFs) are key components of the stroma and display high heterogeneity with multiple subtypes present (inflammatory, myofibroblastic, and antigen presenting). Inflammatory CAFs, which are located away from the cancer cells, are favored under IL‐1/JAK/STAT signaling and secrete tumor‐promoting cytokines. On the other hand, myofibroblastic CAFs, which are located next to the cancer cells, are favored under TGFB signaling and are the primary producers of the extracellular matrix components. Finally, antigen‐presenting CAFs express MHC II molecules to interact directly with immune cells. PDAC is considered an immunologically “cold” tumor as CAFs, tumor‐associated macrophages (TAMs), regulatory T cells (Tregs) facilitate immune evasion and promote tumor growth. Created with BioRender.com.

PDAC is characterized by a unique TME with an extensive desmoplastic stroma that can make up to 90% of the tumor volume.^141^ Cellular crosstalk between neoplastic and non‐neoplastic cells, including fibroblasts, endothelial, immune cells, create an immunosuppressive or “cold” TME in PDAC (Figure 1).^142^ The dense desmoplastic stroma in PDAC is made up of ECM constituents such as collagen secreted by CAFs upon signaling from neoplastic cells.^143^ Interestingly, research has demonstrated that CAFs demonstrate both tumor‐suppressive and tumor promoting properties.^144^ This could be attributed to the recent identification of different subtypes of CAFs (inflammatory, myofibroblastic, and antigen presenting) that exhibit distinctive gene expression profiles and function.^145^ Regardless, the resulting desmoplasia promotes tumor progression by significantly limiting drug penetration and immune cell infiltration. CAFs are further capable of creating an immunosuppressive TME by secreting suppressive factors that recruit immunosuppressive T regulatory cells (Tregs) and myeloid cells and upregulating immune checkpoints on effector T cells.^146^

Subsets of myeloid cells, most notably TAMs and myeloid‐derived suppressor cells (MDSCs), also contribute to the immunosuppressive TME of PDAC.^143^ TAMs can be placed on a spectrum from antitumoral M1 macrophages to protumoral M2 macrophages for simplification.^147^, ^148^ M2 macrophages support an immunosuppressive TME by secreting IL‐6 and TGF‐ß, promoting the differentiation of CD4^+^ T cells into Tregs, and expressing high levels of T cell suppressive ligands such as PD‐L1, which can trigger T cell exhaustion.^143^, ^146^ Distinct from TAMs, MDSCs are immature myeloid cells characterized by the expression of signature markers CD11b, CD33, and CD14 or CD15/66b.^143^ They can be classified into two main populations: polymorphonuclear (PMN)‐MDSCs/granulocytic‐MDSCs and mononuclear MSDCs (M‐MDSCs).^149^ MDSCs, based on the subpopulation they belong in, promote an immunosuppressive TME through a range of different mechanisms.^150^ Multiple key enzymes, including arginase‐1 and indoleamine 2,3‐dioxygenase, are activated, depleting available l‐arginine and l‐tryptophan and causing T cell inhibition.^151^ Similar to TAMs, MDSCs also express inhibitory receptors (e.g., PD‐L1 and CTLA‐4) and recruit Tregs through CD40 engagement to induce T cell exhaustion and tolerance.^151^, ^152^ The high production of reactive oxygen species and nitric oxide by PMN‐MDSCs and M‐MDSCs respectively inhibit the fitness, proliferation, and movement of T cells within the TME.^151^, ^153^

## 3 CURRENT DEBATE ON USE OF NAT

While the benefits of adjuvant therapy after surgical resection have been established through multiple RCTs, several drawbacks of adjuvant therapy have led to a shift toward a neoadjuvant approach after success in other types of cancer.^154^, ^155^, ^156^ RCTs have shown the need for systemic treatment in addition to surgical resection and that those who do not receive chemotherapy have significantly worse prognosis.^157^ The observation arm (without adjuvant chemotherapy) of the CONKO‐001 trial reported a median DFS of only 6.7 months after surgery, and 50% of these patients experienced recurrence or death within 6 months of surgery.^9^, ^16^ Despite the established advantages of chemotherapy, patients often experience disease progression and postoperative complications, precluding them from being treated with adjuvant chemotherapy after surgical resection. Around 45% of patients do not receive adjuvant chemotherapy as a result of clinical deterioration, postoperative complications and morbidity, or early progression of disease.^158^, ^159^ Furthermore, some PDAC tumors may form after a more prolonged precursor lesion stage, and micrometastasis can occur early on in the tumor development stage.^160^ This can be observed as patients often experience local recurrence or metastasis despite a R0 resection; NAT has the potential of eradicating these micrometastases early on prior to surgical resection.^161^ NAT was originally introduced for the potential benefits of tumor downstaging prior to surgical resection, but is now more used for identifying patients with favorable tumor biology that will benefit from surgical resection.^162^ Furthermore, NAT is capable of increasing R0 resections, which clinically associated with improved outcomes of patients following curative‐intent surgery.^163^, ^164^, ^165^, ^166^, ^167^ Ultimately, all these factors play a role in the primary goal of NAT—to reduce risk of recurrence after curative surgery.

However, arguments against NAT and in favor of upfront surgery with adjuvant therapy still exist. Disease progression during NAT leading to prevention of curative‐intent resection is a major concern of oncologists and surgeons alike, with some preferring upfront surgery to ensure that resection is performed.^168^, ^169^ Additionally, administering neoadjuvant chemotherapy potentially reduces patient quality of life and clinical performance due to toxicities, which may complicate surgeries or even preclude patients from undergoing surgical resection.^16^ Particularly, FOLFIRINOX has been particularly noted for its adverse gastrointestinal complications, and patients treated with the combination therapy have increased risks of infections and neuropathy.^11^, ^16^ Finally, administration of NAT increases the risk of intraoperative bleeding and adjacent tissue damage by causing inflammation and fibrosis of the tumor‐surrounding tissue.^170^

## 4 ADMINISTRATION OF NAT BASED ON RESECTABILITY

### 4.1 Locally advanced pancreatic cancer

Staging of the tumor plays a critical role in determining whether the patient should receive NAT or not. NAT has shown to be beneficial for patients with LAPC or BRPC. A study of 415 LAPC patients from 2013 to 2019 showed that surgical resection after NAT is achievable in a highly selected group of patients.^171^ Eighty‐four (20%) of the 415 patients underwent surgical resection after NAT (median duration of 5 months) and had significantly higher median OS (35.3 vs. 16.3 months) and smaller tumor size compared with patients who did not undergo surgical resection.^171^ NAT not only provides better outcomes for patients with LAPC, but it also facilitates more precise selection of patients who will benefit from and should undergo surgical resection.^158^, ^172^ Patients who experience disease progression or no response to neoadjuvant chemotherapy are unlikely to respond favorably to adjuvant chemotherapy as well, thus increasing the risk of recurrence after surgical resection and indicating a more aggressive malignancy. Consequently, these patients who are precluded from surgical resection are most likely spared from an unnecessary and complicated surgical procedure. Several other studies have also reported favorable results after surgical resection following NAT even though imaging post‐NAT shows continued irresectability.^158^, ^173^, ^174^ Tee et al.^175^ found that the 2‐year OS rate of LAPC patients who underwent pancreatectomy with arterial resection following NAT was significantly higher than patients who underwent upfront surgery (62.3 vs. 25.8%, *p* = 0.038). Overall, NAT should be administered for patients with LAPC to not only reduce tumor size but also to help select candidates for resection who do not have disease progression, which is an indicator of a biologically aggressive disease.^158^

### 4.2 Borderline resectable pancreatic cancer

Approximately 30% of PDAC patients have borderline resectable tumors, and current guidelines suggest the use of NAT following the completion of several RCTs.^164^ Jang et al.^176^ reported the first prospective RCT for NAT on BRPC patients. Patients in the experimental arm were treated with four cycles of neoadjuvant gemcitabine‐based chemoradiotherapy followed by surgical resection, while patients in the control arm received upfront surgery followed by four cycles of adjuvant gemcitabine‐based chemoradiotherapy.^176^ Both groups received another four cycles of gemcitabine maintenance chemotherapy. The study found that the neoadjuvant group had significantly better 2‐year survival rate (840.7% vs. 26.1%, hazard ratio HR 1.495, *p* = 0.028) and R0 resection rate (51.8 vs. 26.1%, *p* = 0.004).^176^ The study was terminated due to the statistically significant benefits of neoadjuvant chemoradiation.^176^

In the recently completed phase III PREOPANC‐1 trial, 246 patients with resectable pancreatic cancer or BRPC were randomized to either upfront surgery with adjuvant gemcitabine or neoadjuvant gemcitabine‐based chemoradiotherapy and adjuvant gemcitabine.^177^, ^178^ Long‐term follow‐up showed a significant increase in median OS for patients that underwent NAT compared with upfront surgery (15.7 vs. 14.3 mo, HR 0.73, *p* = 0.025).^178^ Furthermore, R0 resection, which is a prognostic predictor for PDAC, was achieved in 49 out of 68 (72%) of patients who underwent neoadjuvant chemoradiotherapy versus 35 out of 82 (43%) of patients who underwent upfront surgery (*p* < 0.001).^178^ Interestingly, interim analysis of the clinical trial did not yield any significant results, highlighting the need for future RCTs to follow‐up long term to obtain meaningful results. Previous RCTs have also reported the safety and the benefits of NAT against BRPC in terms of R0 resection rate and median OS.^176^, ^179^, ^180^, ^181^

### 4.3 Resectable pancreatic cancer

While the use of NAT for BRPC is supported by other RCTs, the evidence is not as clear for patients with resectable tumors at presentation. Some studies support the use of NAT against resectable tumors.^16^ The phase II‐III Preop‐2/JSAP‐05 trial in Japan compared the effects of NAT with gemcitabine and S‐1 chemotherapy versus upfront surgery, both of which were followed by adjuvant S‐1 chemotherapy on resectable patients.^182^ The RCT demonstrated superior survival, with a median OS of 37 versus 27 months, for the group who underwent NAT (HR 0.72, 95% confidence interval CI 0.55–0.94, *p* = 0.015).^182^ However, administering NAT and delaying surgery may result in losing the opportunity for surgical resection, particularly for patients with resectable tumors at presentation, as a result of disease progression during NAT.^177^ The hazard ratio of 0.73 in favor of NAT in the PREOPANC‐1 trial was primarily driven by patients with BRPC (HR 0.67, *p* = 0.045) rather than with resectable tumors (HR 0.79, *p* = 0.23).^177^, ^178^ Furthermore, results of the SWOG‐S1505 trial showed that patients with resectable disease who underwent NAT (either mFOLFIRNOX or gemcitabine plus nab‐paclitaxel) did not show superior results compared with patients with similar disease progression who underwent surgery first historically.^177^, ^183^ A meta‐analysis of 7 RCTs that studied NAT on resectable pancreatic cancer or BRPC concluded that more evidence is needed to demonstrate whether patients with resectable tumors should be treated with NAT as opposed to upfront surgery.^184^ The current standard of care based on the 2022 NCCN guidelines for resectable PDAC is upfront surgery and adjuvant treatment.^185^ The guidelines advise the use of NAT when high risk features such as significantly elevated carbohydrate antigen (CA) 19‐9, large primary tumor or lymph nodes, suspicions of advanced or metastatic disease are observed.^185^ A list of completed RCTs on the use of NAT for patients with resectable pancreatic cancer or BRPC are summarized in Table 1.

**TABLE 1:** Completed randomized clinical trials comparing neoadjuvant therapy and upfront surgery for resectable and borderline resectable pancreatic cancer

Trial	Accrual years	Number of patients	Tumor staging	Treatment regimen (cycles)	Comparator (cycles)	Median OS (months)	Resection rate	R0 resection rate
Golcher et al.^180^ NCT00335543	2003–2009	73	Resectable	Neoadj. gemcitabine + cisplatin‐based CRT 54 Gy + adj. gemcitabine (6)	Adj. gemcitabine	17.4 vs. 14.4 (*p* = 0.96)	58 vs. 70% (*p* = 0.31)	52 vs. 48% (*p* = 0.81)
PACT‐15^179^ NCT01150630	2010–2015	88	Resectable	Periop. PEXG (3+3)	Adj. PEXG (6) Adj. gemcitabine (6)	38.2 vs. 26.4 vs. 20.4	84 vs. 90 vs. 85%	63 vs. 27 vs. 37%
Jang et al.^176^	2012–2014	50	BPRC	Neoadj. gemcitabine CRT 54 Gy (4) + adj. gemcitabine (4)	Adj. gemcitabine CRT (4) + Adj. gemcitabine (4)	21.0 vs. 12.0 (*p* = 0.028)	63.0 vs. 78.3%	51.8 vs. 26.1% (*p* = 0.064)
Preop‐02/JSAP‐05^186^ UMIN000009634	2013–2016	360	Resectable/BRPC	Neoadj. S‐1 + gemcitabine (2) + adj. S‐1 (6 mo.)	Adj. S‐1 (6 mo.)	36.7 vs. 26.6 (*p* = 0.015)	NR	NR
PREOPANC‐1^178^ NL3525	2013–2017	246	Resectable/BRPC	Neoadj. gemcitabine CRT 36 Gy (3) + adj. gemcitabine (4)	Adj. gemcitabine (6)	15.7 vs. 14.3 (*p* = 0.025)	61 vs. 72%	72 vs. 43% (*p* < 0.001)
NEPAFOX^187^ NCT02172976	2011–2014	40	Resectable/BRPC	Periop. FOLFIRINOX (4‐6 + 4–6)	Adj. gemcitabine (6)	10.0 vs. 25.7	57.9 vs. 85.7%	45 vs. 72%
ESPAC‐5F^187^	2014–2018	88	BRPC	B: Neoadj. GnP (2) C: Neoadj. mFOLFIRINOX (4) D: Neoadj. capecitabine CRT 50.4 Gy All arms were given adj. 5‐Fu or adj. gemcitabine (6)	Adj. 5‐Fu or adj. gemcitabine (6)	NR	55 (neoadj.) vs. 66% (upfront surgery)	23 (neoadj.) vs. 15% (upfront surgery) (*p* = 0.721)

CRT, chemoradiotherapy; BPRC, borderline resectable pancreatic cancer; neoadj., neoadjuvant; adj., adjuvant; periop., perioperative; FOLFIRINOX, folinic acid + irinotecan + oxaliplatin + leucovorin; mFOLFIRINOX, modified FOLFIRINOX; GnP, gemcitabine plus nab‐paclitaxel; PEXG, cisplatin + epirubicin + capecitabine + gemcitabine; NR, not reported.

### 4.4 Late‐stage PDAC

**FIGURE 2:**
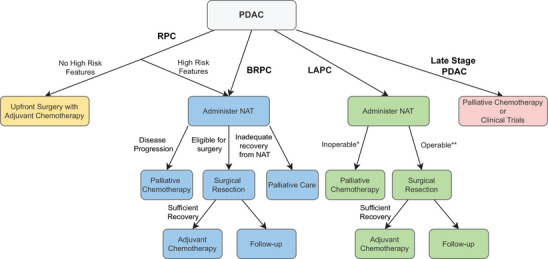
Flow diagram for the use of neoadjuvant therapy against PDAC. *Inoperable includes cases of poor response to NAT, disease progression, or insufficient recovery from NAT. **Operable includes cases of tumor shrinkage and sometimes stable disease since radiographic restaging often does not reflect whether the tumor can be resected. Created with Draw.io.

Patients with late‐stage PDAC, which refers to T_2‐4_N_0_M_0_ and nodal or distal metastatic PDAC, are inoperable, and palliative therapy had been the only available treatment option historically.^188^, ^189^ Current guidelines recommend (m)FOLFIRINOX or gemcitabine with nab‐paclitaxel (GnP) for patients with better performance status (ECOG 0–1) and single agent gemcitabine with or without a second agent for patients with worse performance status (ECOG 2–3).^190^ Recent developments in novel treatments like targeted therapy and immunotherapy have offered many of these inoperable patients another avenue of treatment and a chance to participate in ongoing clinical trials for new agents. Furthermore, germline and somatic testing, suggested by current NCCN guidelines, provide the opportunity for more personalized medicine and a wider range of options for these patients.^190^, ^191^ Figure 2 summarizes in a flow chart the current guidelines on the use of NAT depending on resectability.

## 5 ADMINISTRATION OF NAT BASED ON BIOMARKERS

Recent studies have identified possible biomarkers that have diagnostic, prognostic, or predictive value for PDAC.^192^ Another approach to determine whether patients should receive NAT is examining different predictive biomarkers that have been associated with either better or worse response to NAT. Given the genetic and molecular heterogeneity of PDAC, patients and tumor response to NAT differ greatly and provides the opportunity to personalize treatment in this era of precision medicine.^193^ A subset of PDAC patients does not respond to NAT and may benefit more from upfront surgery, and recent research have focused on trying to identify predictive biomarkers for this subset of patients. Tsai et al.^194^ conducted the first phase II trial of using molecular profiling of pretreatment endoscopic ultrasound‐guided fine‐needle aspiration (EUS‐FNA) biopsies to individualize NAT regimen. Six biomarkers (*TYMS, ERCC1, RRM1, SPARC, TOP1, hENT1*) from the EUS‐FNA were used to determine the course of NAT for 130 resectable and borderline resectable patients: 80% were treated with 5‐Fu based chemotherapy and 20% with gemcitabine‐based chemotherapy.^194^ The study reported a median OS of 38 months, 5‐year survival rate of 34%, and resection rate and R0 resection rate of 82 and 81%, respectively.^194^ The high rate of resection suggests that molecular profiling of pretreatment samples can individualize treatment and improve efficacy.^194^

In another study, Sahni et al.^195^ conducted SWATH‐MS proteomic analysis on samples from resected tumors following NAT to identify upregulated protein biomarkers predictive of NAT response. The study identified that the upregulation of four proteins (*GRP78*, *CADM1*, *PGES2*, and *RUXF*) was highly predictive for chemoresistance.^195^ Upregulation of *TMED2*, *AGR2*, *JTB*, and *CADM1* were also found to be overexpressed in poor responders, but future studies on the levels of these biomarkers in the plasma/serum are needed to determine the ability of these biomarkers to predict NAT response.^195^

More recently, Shoucair et al.^15^ used targeted RNA sequencing of frozen FNA biopsy specimens to identify genes with atypical expression that were associated with patient response to NAT. In a discovery cohort of 23 patients, the study found that that lower expression of matrix metalloproteinase 7 (*MMP7*) was associated with more favorable pathologic response, which is a strong surrogate for outcomes of PDAC patients.^15^ The finding was confirmed in a validation cohort of 80 patients: patients with negative *MMP7* immunohistochemistry staining were significantly more likely to have a more favorable pathologic response to NAT (odds ratio 21.25, 95% CI 6.19–72.95, *p* = 0.001). These results show great promise for use of RNA sequencing and proteomic analysis to identify targets that can predict NAT response. Ultimately, these predictive biomarkers could be incorporated into deciding course of treatment after research with a larger sample size obtains significance.

Moving forward as more biomarkers are discovered are verified, specific treatment plans and regimens must be developed for these special groups of patients to effectively provide care. For example, just as patients with lower expression of *MMP7* are predicted to respond well to NAT, a new treatment plan with either new drugs or steps is required for patients who have higher expression of *MMP7*. Then RCTs must be carried out to validate the alternative treatment plan for these specific groups of patients. Ultimately, while the use of biomarkers like *MMP7* is not practical as of right now due to the resources and time required to complete RNA sequencing or proteomic analysis, these methods provide great opportunities to individualize and enhance treatments for patients in the future.

## 6 ASSESSMENT OF PDAC RESPONSE TO NAT AND PROGNOSTIC MEANING

### 6.1 Imaging response

Once the decision to administer NAT has been made, it is critical to be able to accurately assess patient response to NAT as it offers meaningful prognostic value and can guide future course of treatment. However, methods for assessment have not been standardized. Staging and restaging of PDAC have been traditionally done based on a radiographic response, most commonly with multidetector computed tomography (CT), positron emission tomography (PET) with CT, magnetic resonance imaging, and endosonography.^196^ The 2022 NCCN guidelines recommend PET/CT scan for detection of extra pancreatic metastases in the case of high risk patients as PET offers a more in depth look at the morphological and metabolic activity of the lesions.^197^ However, PET/CT should only be used in conjunction with high‐quality contrast‐enhanced CT, and not to replace it.^198^ Having said that, assessing tumor response to NAT and determining resectability with only imaging is insufficient.^196^ A recent systemic review on BRPC and LAPC reported that most patients had stable disease after NAT and did not experience change in tumor size, volume, or density; only a fraction of patients were observed to have a partial response with tumor size reduction and a decrease in standardized uptake value on the PET scan.^199^ The imaging criteria were found to be conflicting with the histopathological tumor regression grading, underlining the inadequacy of measuring NAT response with imaging only.^199^ Furthermore, determining tumor response and resectability following NAT using imaging may be tricky as inflammation caused by NAT can mimic a solid tumor.^200^

### 6.2 CA 19‐9

Biomarkers have also been used to supplement imaging assessment of NAT response. Currently, CA 19‐9 is the most widely used and only Food and Drug Administration‐approved biomarker to manage PDAC.^201^ Multiple studies have demonstrated the prognostic value of measuring CA 19‐9 before and throughout treatment for PDAC.^202^, ^203^, ^204^ An elevated baseline or preoperative CA 19‐9 level has been associated with a poor prognosis for PDAC with a shorter median survival; similarly, an increase in CA 19‐9 levels throughout treatment is also a sign of disease progression and predictor of worse outcomes. A study with 93 and 41 PDAC patients in the training and validation set respectively reported that CA 19‐9 elevation is an early and reliable sign for PDAC recurrence: 2.45 times elevated CA 19‐9 values showed recurrence with 90% positive predictive rate.^205^ In terms of NAT, CA 19‐9 levels post‐NAT have been found to be predictive of tumor size reduction and survival. In a study of 250 patients, a CA 19‐9 response ≥85% was a strong independent predictor of tumor size reduction ≥25% (HR 2.40, *p* = 0.007) and improved survival (HR 0.47, *p* = 0.007).^206^ Results from Heger et al.’s^207^ retrospective study showed that CA 19‐9 levels after neoadjuvant FOLFIRINOX more accurately predicts resectability and survival than dynamic values. Combined, this evidence suggests the need to incorporate CA 19‐9 response as an endpoint for NAT and decision making in resectability after NAT.^206^, ^207^ Having said that, CA 19‐9 levels can be difficult to interpret when the patient has a biliary obstruction or presents with a normal CA 19‐9 level initially.^196^ Other biomarkers such as carcinoembryonic antigen and CA 125 have been found over the past decade to complement serum CA 19‐9 levels for diagnosis and prognosis.^208^

### 6.3 Circulating tumor cells and circulating tumor DNA

Recently, research has focused on the role of circulating tumor cells (CTCs) and circulating tumor DNA (ctDNA) from liquid biopsies as biomarkers for the screening and monitoring of PDAC patients.^209^, ^210^, ^211^, ^212^, ^213^ Effenberger et al.^214^ showed that CTC status affects the outcome of PDAC patients independent of other risk factors and can stratify patients based on OS and progress‐free survival (PFS). CTCs were identified in 78% of patients and were correlated with increasing stage (*p* < 0.001) in a study by Court et al.^215^ Of the 53 patients who underwent surgical resection in Court et al.’s^215^ study, those who eventually experienced occult disease has significantly higher number of CTCs (*p* < 0.0001). Furthermore, Court et al. found that CTCs were a multivariate predictor of OS (HR 1.38, 95% CI 1.01–1.88, *p* = 0.040) and a univariate predictor of recurrence‐free survival postsurgery (HR 2.36, 95% CI 1.17–4.78, *p* = 0.017).^215^ Similarly, another study with 112 patients found that higher levels of percent‐ctDNA were a multivariate, independent prognostic factor for OS (HR 4.35, 95% CI 1.84–10.24, *p* = 0.001) in pancreatic cancer.^216^ All this evidence show promise for the use of CTC and ctDNA from liquid biomarkers to assess patient status.

However, there exists several issues with using CTC and ctDNA for screening, diagnostic, and prognostic purposes that have yet to be overcome.^217^ First, a meta‐analysis on ctDNA in pancreatic cancer reported a low pooled sensitivity of only 0.64, which could be attributed to the inadequate amount of ctDNA released in the plasma as a result of low tumor necrosis in early PDAC.^218^ This low sensitivity makes it difficult for the use of ctDNA in the screening of PDAC. A more recent meta‐analysis calculated a pooled specificity of ctDNA of 0.92 in PDAC, which is only marginally better than the specificity of CA 19‐9.^217^, ^218^ While the theoretical benefits of using ctDNA as a new marker have yet to make a significant clinical impact due to technological limitations, several ongoing studies are currently exploring their use in a clinical setting. An ongoing multicenter study (NCT04246203) conducted by the University of Munich is monitoring and comparing disease progression between patients with and without the presence of detectable ctDNA in a liquid biopsy. CAPS5 (NCT02000089), the current CAPS program, may have also incorporated surveillance of circulating epithelial cells from pancreatic duct fluid as a primary outcome.^219^ Overall, the use of CTC and ctDNA for diagnosing and monitoring PDAC is extremely promising as technologies become more developed.

### 6.4 Histopathologic response

Another method to assess patient tumor response to NAT is through a histopathologic approach. Multiple grading systems of tumor regression/response in posttreatment pancreatectomy exist for PDAC, including the Evans, White, Ishikawa, and College of American Pathologists (CAP) system.^220^, ^221^, ^222^ The CAP system, one of the more commonly used systems, has four scoring tiers: score 0, complete response; score 1, near complete response; score 2, partial response; score 3, poor or no response.^223^ While the CAP system has been criticized for the lack of difference in survival between score 2 and 3 patients, Maeda et al.^6^ reported that scores 2 and 3 have different prognostic meanings for patients treated with neoadjuvant chemotherapy versus neoadjuvant chemoradiotherapy. Despite scores 2 and 3 not having a significantly different OS for neoadjuvant chemoradiotherapy, a CAP score of 2 was found to be in an independent prognostic factor for improved OS and DFS for neoadjuvant chemotherapy.^6^

A pathological complete response (pCR) is defined as the absence of any observable cancer cells in the pancreas or lymph node on final pathology or a CAP score of 0.^224^ Achieving pCR after NAT and resection is a prognostic factor for improved outcomes, while long‐term survival is less common if patients have a poor histopathologic response following NAT and pancreatectomy.^196^, ^225^, ^226^ In a retrospective study of 186 BRPC/LAPC patients who underwent NAT and pancreatectomy at Johns Hopkins Hospital, 19 (10%) achieved pCR.^226^ Achieving pCR was found to be an independent prognostic factor for DFS (HR 0.45, 95% CI 0.22–0.93, *p* = 0.030) and OS (HR 0.41, 95% CI 0.17–9.97, *p* = 0.044).^226^ However, pCR is rarely achieved in PDAC, with a reported observance of 4–15%.^226^, ^227^, ^228^, ^229^ A follow‐up study at Johns Hopkins Hospital found that only 30 out of 331 patients achieved pCR after NAT and pancreatectomy.^224^ Interestingly, even in the rare occurrence of achieving pCR, a portion of these patients develop recurrence despite the lack of any observable cancer cells.^224^ Out of the 29 eligible pCR patients, 14 (48%) had recurrence with nine out of the 14 dead at the last follow‐up of the study.^224^ No clinicopathological factor, including the NAT regimen, radiation modality, and location of recurrence, was significantly associated with OS, DFS, or PFS, highlighting the potential limits of a histopathological approach.^224^

Given the variable outcomes despite achieving pCR, Yin et al.^200^ incorporated liquid biopsy with next‐generation sequencing (NGS) of resected specimen to introduce the new concept molecular complete response (mCR). mCR is defined as the absence of tumor‐related mutations in both resected specimen and plasma (CTC and ctDNA).^200^ Thirty‐six patients who achieved pCR were selected from a pool of 479 patients who underwent NAT.^200^ Six patients were identified as mCR, while 15 patients who were positive for either tumor‐related mutations in resected samples or plasma (CTC or ctDNA) were classified as non‐mCR.^200^ Although no statistical significance on patient outcome could be achieved due to the small sample size, this study was the first to report the presence of CTC and ctDNA in patients who achieved pCR after NAT and highlights the value of liquid biopsies in prognostic assessment and outcome prediction.^200^ As future research increases the sensitivity of these biomarkers from liquid biopsies, they can be incorporated into patient assessment prior to and after NAT treatment to determine resectability and guide future adjuvant therapeutic choices.

Ultimately, we recommend a combination of established methods—imaging, CA 19‐9, and histopathological—to assess patient response to NAT, reevaluate resectability, and to eventually determine a plan of care for patients going forward. Other more novel methods such as biomarkers and molecular analysis can be incorporated, but more research is required to validate these findings. Most studies suggest that surgical resection be performed 4–8 weeks after completion of NAT if the patient is a candidate for surgery.^170^

## 7 SELECTING PDAC NAT REGIMENS AND THE USE OF RADIOTHERAPY

Over the past few decades, advances in chemotherapy have increased the efficacy of systemic treatment for PDAC. Particularly, the developments of combination therapy such as (m)FOLFIRINOX or gemcitabine combination therapies have shown enhanced tumor‐reducing effects and increased OS compared with gemcitabine or fluorouracil monotherapies in the adjuvant fashion.^11^, ^230^, ^231^, ^232^ However, the optimal NAT regimen has not been fully established yet and more RCTs directly comparing different treatments are required to determine the advantages and disadvantages of each regimen. Currently, no specific neoadjuvant regimen is recommended based on evidence from RCTs, but (m)FOLFIRINOX or GnP are the most commonly used in clinical trials.^16^ A single‐arm phase II study completed at the Massachusetts General Hospital treated 48 BPRC patients with eight cycles of neoadjuvant FOLFIRINOX followed by personalized chemoradiotherapy.^233^ Forty‐four patients (92%) proceeded to chemoradiotherapy, and surgical resection was performed on 32 patients (67%).^233^ Median OS was 38 months, and 2‐year OS rate was 56%, demonstrating the safety and efficacy of neoadjuvant FOLFIRINOX followed by radiotherapy.^233^ In the ALLIANCE A021501 trial, patients with BRPC were either treated with eight cycles of neoadjuvant mFOLFIRINOX or seven cycles of neoadjuvant mFOLFIRINOX with stereotactic body radiation therapy.^234^ Initial results showed an 18‐month survival rate of 67.9% (95% CI: 5.46–78.0) in the group that received neoadjuvant chemotherapy only, establishing mFOLFIRINOX as a viable regimen for NAT.^235^ Other RCTs that utilized gemcitabine‐based NAT, including the Dutch PREOPANC‐1, have also demonstrated the safety and efficacy of gemcitabine‐based NAT.^176^, ^178^, ^179^, ^180^

### 7.1 Retrospective studies comparing NAT regimens

A number of retrospective studies and meta‐analyses have been completed to determine the optimal NAT regimen.^236^, ^237^, ^238^ Tang et al.^238^ conducted a meta‐analysis that included eight retrospective studies to compare head‐to‐head neoadjuvant FOLFIRINOX versus GnP for localized pancreatic cancer. Three out of the eight included studies reported a significantly prolonged OS for FOLFIRINOX, while the remaining five demonstrated a similar efficacy in terms of OS.^238^ Furthermore, a sub‐group analysis adjusted for age also showed significantly longer OS for FOLFIRINOX when compared with GnP.^238^ Yet despite the 1–3‐year survival rates being higher for patients treated with FOLFIRINOX, the 5‐year survival rates were comparable between patients who were treated with FOLFIRINOX versus GnP.^238^ In terms of perioperative parameters, the meta‐analysis found that patients with either treatment had statistically similar resection rates and R0 resection rates, but patients who were treated with FOLFIRINOX had a significantly lower perineual invasion rate, which may contribute to its longer OS.^238^ Ultimately, Tang et al.^238^ concluded that FOLFIRINOX is noninferior compared with GnP for patients who are capable of receiving FOLFIRINOX. A more recent single institution retrospective study comparing FOLFIRINOX and GnP for LAPC demonstrated that NAT with FOLFIRINOX is associated with more favorable results versus GnP as well.^237^ A better OS was observed in the FOFLIRINOX group (85.1 vs. 54.3 weeks); the authors suggested that this may be a result of the higher rates of conversion/radical surgery for patients treated with FOLFIRINOX (41.8 vs. 22.2%).^237^ However, FOLFIRINOX has been associated with higher risk of adverse events, particularly in the elderly, so patient condition must also be taken into account in addition to the efficacy of the chemotherapy agents when deciding the treatment course.^11^, ^239^

### 7.2 Completed and ongoing RCTs comparing NAT regimens

Despite the results from these retrospective studies, RCTs that directly compare different neoadjuvant chemotherapeutic regimens for PDAC are required to provide conclusive evidence. Table 2 shows four RCTs that compare different neoadjuvant chemotherapeutic and novel immunotherapeutic agents. Notably, the SWOG S1505 trial randomized patients with resectable PDAC to either perioperative (three cycles neoadjuvant and three cycles adjuvant) FOLFIRINOX or GnP. The researchers found no significant difference between the 2‐year OS rate, the primary endpoint, between the two groups (41.6 vs. 48.8%), nor statistically significant improvement compared with the a priori 2‐year OS rate of 40%.^240^ And while the SWOG S1505 trial demonstrated safety and relatively high resectability rates of both neoadjuvant chemotherapeutic regimens, it found no significantly improved median OS (22.4 vs 23.6 months) of either regimen compared with historical standards.^240^

**TABLE 2:** Completed clinical trials comparing different neoadjuvant therapy regimens

Trial	Accrual years	Number of patients	Tumor staging	Treatment regimen (cycles)	Comparator (cycles)	Median OS (months)	Resection rate	R0 resection rate
SWOG S1505^242^ NCT02562716	2010–2015	102	Resectable	Periop. FOLFIRINOX (3 + 3)	Periop. GnP (3 + 3)	22.4 vs. 23.6	73 vs. 70%	85 vs. 85%
ALLIANCE A021501^243^ NCT02839343	2012–2016	126	BRPC	Neoadj. FOLFIRINOX (8) + adj. FOLFOX (4)	Neoadj. FOLFIRINOX (8) + neoadj. RT + adj. FOLFOX	17.1 vs. 31.0	51 vs. 58%	25 vs. 42%
Laheru et al. Johns Hopkins^244^ NCT00727441	2003–2015	87	Resectable	Periop. GVAX (1 + 5) + cyclophosphamide (A) IV or (B) oral + adj. CRT	(C) Periop. GVAX (1 + 5) + adj. CRT	15.4 (A) vs. 16.5 (B) vs. 34.2 (C)	86 vs. 87 vs. 90%	78 vs. 67 vs. 64%
NEOLAP^245^ NCT02125136	2014–2018	168	LAPC	Neoadj. GnP (4) + adj. GnP (3) if R0/R1 resection	Neoadj. GnP (2) + neoadj. FOLFIRINOX (4) + adj. GnP (3) if R0/R1 resection	18.5 vs. 20.7 *p* = 0.53	35.9 vs. 43.9% *p* = 0.38	No significant difference

CRT, chemoradiotherapy; RT, radiotherapy; LAPC, locally advanced pancreatic cancer; BPRC, borderline resectable pancreatic cancer; neoadj., neoadjuvant; adj., adjuvant; periop., perioperative; FOLFIRINOX, folinic acid + irinotecan + oxaliplatin + leucovorin; FOLFOX, folinic acid + oxaliplatin + leucovorin; GnP, gemcitabine plus nab‐paclitaxel; GVAX, granulocyte‐macrophage colony‐stimulating factor (GM‐CSF)‐secreting allogeneic PDAC vaccine; IV, intravenous.

Several other RCTs that are investigating the optimal neoadjuvant chemotherapeutic and immunotherapeutic regimen are summarized in Table 3. In the ongoing multicenter phase III PREOPANC‐2 RCT, patients with resectable pancreatic cancer or BPRC are randomized into two groups. The first group receives eight cycles of neoadjuvant FOLFIRINOX followed by surgical resection without adjuvant therapy; the second group receives three cycles of gemcitabine with hypofractionated radiotherapy (36 Gy in 15 cycles) during the second cycle followed by surgical resection and four cycles of adjuvant gemcitabine. The 2019 ASCO guidelines suggests a total of 6 months of chemotherapy, taking into consideration both neoadjuvant and adjuvant chemotherapy.^241^

**TABLE 3:** Ongoing randomized clinical trials on neoadjuvant therapy for PDAC

Trial	(Target) sample size	Tumor staging	Treatment regimen (cycles)	Comparator (cycles)	Primary outcome	Status
NorPACT‐1^246^ NCT02919787	140	Resectable	Neoadj. FOLFIRINOX (4) + adj. gemcitabine/capecitabine (4)	Adj. gemcitabine (6)	OS 18 months after randomization	Results pending
PANDAS‐PRODIGE 44^247^ NCT02959879	90	BRPC	Neoadj. mFOLFIRINOX + capecitabine CRT 50.4 Gy + adj. gemcitabine or mLV5Fu	Neoadj. mFOLFIRINOX + adj. gemcitabine or mLV5Fu	R0 resection rate	Recruiting
PREOPANC‐2^245^ NTR7292	368	Resectable/BRPC	Neoadj. FOLFIRINOX (8)	Neoadj. gemcitabine‐based CRT 36 Gy (3) + adj. gemcitabine (4)	OS	Results pending
ALLIANCE A021806^248^ NCT04340141	352	Resectable	Periop. mFOLFIRINOX (8 + 4)	Adj. mFOLFIRINOX (12)	OS	Recruiting
UVA‐PC‐PD101^249^ NCT02305186	68	Resectable/BRPC	Neoadj. Pembrolizumab + neoadj. capecitabine‐based CRT	Neoadj. capecitabine‐based CRT	Number of TILs, toxicity	Recruiting
NEONAX^250^ NCT02047513	127	Resectable	Periop. GnP (2 + 4)	Adj. GnP (6)	DFS	Results pending
PREOPANC‐3^251^ NCT04927780	378	Resectable	Periop. mFOLFIRINOX (8 + 4)	Adj. mFOLFIRINOX (12)	OS	Recruiting
Zheng et al.^252^ NCT03727880	36	Resectable	Neoadj. chemotherapy (2) + neoadj. pembrolizumab (2) + defactinib (6 weeks) + adj. chemotherapy + adj. pembrolizumab (8)	Neoadj. chemotherapy (2) + neoadj. pembrolizumab (2) + adj. chemotherapy + adj. pembrolizumab (8)	Rate of pathologic complete response	Recruiting

CRT, chemoradiotherapy; BPRC, borderline resectable pancreatic cancer; neoadj., neoadjuvant; adj., adjuvant; periop., perioperative; FOLFIRINOX, folinic acid + irinotecan + oxaliplatin + leucovorin; mFOLFIRINOX, modified FOLFIRINOX; GnP, gemcitabine plus nab‐paclitaxel; mLV5Fu, modified LV5Fu (folinic acid + bolus fluorouracil + infusional fluorouracil).

### 7.3 Patient performance status and NAT regimen

In addition to the molecular basis of disease and the efficacy of chemotherapy regimens, the optimal NAT regimen is also dependent on the performance status of patients. FOLFIRINOX and mFOLFIRINOX should be limited to patients with ECOG 0–1, while GnP is acceptable for patients with ECOG 0–2 according to the 2022 NCCN guidelines.^197^ 5‐Fu + leucovorin + liposomal irinotecan is reasonable for patients with ECOG 0–2 if patients have not been previously treated with irinotecan.^197^ Furthermore, comorbidities should be incorporated into the decision making concerning the neoadjuvant regimen and the decision to offer surgery and radiotherapy on a case‐by‐case basis, particularly for older adults.^253^ Gemcitabine or fluorouracil should be considered for patients with an ECOG of 2 or comorbidities that preclude them from more aggressive regimens.^254^

### 7.4 Neoadjuvant radiotherapy

The use of radiotherapy neoadjuvant style also remains a controversial issue. RCTs such as PREOPANC‐1 and Jang et al.^178^ have successfully showed the advantages of neoadjuvant chemoradiotherapy compared with upfront surgery for patients with BRPC and LAPC. This conclusion is supported by other retrospective studies.^255^ Yet, due to the initial results of the ESPAC‐1 trial showing a significant survival benefit of adjuvant chemotherapy but negative effects of adjuvant radiotherapy, neoadjuvant radiotherapy use has been minimized in Europe despite still being commonly used in the United States.^158^ Katz et al.^235^ also reported in an abstract report of the ALLIANCE A021501 trial that no improvement in OS or R0 resection rate were found with the addition of stereotactic body radiation therapy. Furthermore, ESPAC‐5F trial demonstrated that neoadjuvant mFOLFIRINOX or gemcitabine plus capecitabine resulted in similar OS durations, and significantly better than neoadjuvant 50.4‐Gy capecitabine‐based chemoradiotherapy.^256^ These clinical trials along with other studies question whether the addition of radiotherapy in NAT is beneficial or may actually be detrimental.^4^, ^177^, ^257^ Consequently, given the lack of conclusive evidence on the effects of radiotherapy in NAT, we suggest that the use of radiotherapy in NAT should be limited to RCTs only.

## 8 THE CASE FOR AND AGAINST ADJUVANT THERAPY POST‐NAT AND RESECTION

While previous RCTs have clearly demonstrated the benefits of adjuvant therapy postresection, the use of adjuvant therapy after NAT remains controversial as well. No RCT has been done on the use of adjuvant therapy following NAT and surgical resection, with other retrospective studies yielding conflicting results.^258^, ^259^, ^260^, ^261^, ^262^, ^263^, ^264^

Swords et al.^259^ investigated how adjuvant chemotherapy affects patient outcome after neoadjuvant chemotherapy by looking at 4187 patients in the National Cancer Data Base (NCDB). The study found that adjuvant chemotherapy was only associated with improved survival in patients with a lymph node ratio between 0.01 and 0.14.^259^ However, a similar retrospective study by de Geus et al.^263^ reported that additional postoperative chemotherapy following NAT and pancreatectomy was not associated any survival benefits, even in patients with positive lymph nodes or positive resection margins.

On the other hand, a single institution study by Perri et al.^260^ found that postoperative therapy following NAT and pancreatectomy was significantly associated with a longer recurrence‐free survival (HR 0.55, 95% CI 0.29–0.96, *p* = 0.04) but only marginally associated with a longer median OS (HR 0.55, 95% CI 0.29–1.01, *p* = 0.05). The latest multicenter retrospective study conducted by Kamarajah et al.^264^ in 2021 included 6560 patients in the NCDB who underwent surgical resection following neoadjuvant chemotherapy. After propensity score matching, 2016 patients with and 2016 patients without adjuvant chemotherapy, all following neoadjuvant chemotherapy, were included in the analysis.^264^ Patients who received adjuvant chemotherapy had a significantly improved median OS (29.4 vs. 24.9 mo, *p* < 0.001).^264^ Improved median OS for patients who received adjuvant chemotherapy following neoadjuvant chemotherapy as opposed to only neoadjuvant chemotherapy was observed regardless of nodal status, resection margins, and the use of neoadjuvant radiotherapy.^264^ The results of Kamarajah et al.’s study has by far the largest sample size and generalizability, supporting the current ASCO and NCCN guidelines of completing additional adjuvant chemotherapy in patients following NAT.^185^, ^264^, ^265^ In addition to validating the benefits of completing adjuvant therapy following NAT, future RCTs should also look into factors such as dosage, number of cycles, and whether the chemotherapy agent should be changed between neoadjuvant and adjuvant therapy as no definitive conclusion has been reached.

## 9 NEXT GENERATION SEQUENCING AND TARGETED THERAPY

Despite making up the backbone of PDAC treatment, the effects of conventional chemotherapy have been limited due to development of chemoresistance, which can occur as a result of the dense desmoplastic stroma inhibiting drug delivery and interactions among cells in the TME.^266^, ^267^ Thus, there is an urgent need for alternatives that improve drug delivery and/or target different mechanisms of PDAC pathogenesis. Better understanding of tumor biology and genetics recently have sparked the development of novel targeted therapies that offer more strategies against specific vulnerabilities.

Several large‐scale studies using NGS have uncovered the complex mutational landscape of PDAC, identifying four common driver genes and approximately 20 less common genes that are mutated in PDAC, some of which could serve as therapeutic targets.^268^, ^269^, ^270^, ^271^ A retrospective analysis of the Know Your Tumor Program, which offers patients commercially available multiomic profiling and molecularly personalized therapy options, reported that while 28% of patients had genetic alterations with an actionable targeted therapy, only 7% of these patients received a matched, targeted therapy.^47^, ^272^ This goes to show the possible impact that NGS and targeted therapy can have and the need to make these options more widely available.

### 9.1 Targeting germline mutations

Several studies have combined to show that cancer predisposing germline mutations, which are directly passed down from parents to children, are present in 3.8–9.7% of PDAC patients.^273^, ^274^ These mutations predispose individuals for several hereditary cancers, such as ovarian and breast cancer (*BRCA1, BRCA2, ATM, PALB2*), atypical multiple mole melanoma (*CDKN2A*), Peutz‐Jeghers (*STK11*), and so on.^274^, ^275^, ^276^, ^277^ The NCCN recommends that all patients diagnosed with PDAC to undergo germline mutation testing regardless of stage and family history.^197^ Additionally, genetic testing for at‐risk individuals with family history of cancers can help enhance screening measures and reach asymptomatic individuals earlier on in the disease.^47^

Mutations that negatively affect DNA damage repair genes, including *BRCA1, BRCA2, ATM, and PALB2*, are one of the better studied group of mutations.^278^, ^279^ These genes encode for key proteins in homology‐directed repair (HDR), which is an error‐free repair system for DNA double strand breaks (DSB).^280^, ^281^ When loss of function mutations occur in one or more of these genes, HDR is impaired, and cells become more reliant on nonhomologous end joining and other DSB repair systems, which are more error prone.^282^ Previous studies have reported that HDR‐mutated ovarian and breast cancers are more susceptible to drugs that induce DSB, such as platinum‐based chemotherapy.^283^, ^284^, ^285^ In 2018, Blair et al.^286^ reported that within a group of *BRCA1/2*‐mutated PDAC patients, platinum‐based adjuvant chemotherapy was associated with better outcomes compared with nonplatinum‐based adjuvant chemotherapy and no adjuvant chemotherapy (31.0 vs. 17.8 vs. 9.3 mo, *p* < 0.001). Furthermore, in a retrospective study of 50 HDR‐mutated patients out of 262 total patients, Park et al.^287^ demonstrated patients with HDR mutations had better PFS compared with non‐HDR‐mutated patients when treated with first‐line platinum therapy, but not when the HDR‐mutated patients with nonfirst line platinum therapy. Finally, a recently completed phase II study in patients with demonstrated that gemcitabine + cisplatin treatment is an effective treatment against *BRCA1/2‐* and *PALB2*‐mutated PDACs.^288^ Based on these evidence, platinum‐based chemotherapy, such as gemcitabine + cisplatin or FOLFIRINOX, should be considered as first‐line treatments for patients with HDR mutations.

Poly (ADP‐ribose) polymerase (PARP) inhibitors (PARPi) are another group of drugs that have shown great promise against HDR‐mutated PDAC.^289^, ^290^ PARP enzymes play a role in repairing DNA single strand breaks (SSB) through base excision repair. PARPi prevents repair of SSB by trapping PARP enzymes on DNA, creating a stall in the replication fork and converting the SSB into a DSB.^282^, ^289^ While normal cells without HDR mutations are able to repair the induced DSB via HDR, HDR‐deficient cells are unable to repair the DSB in an error‐free way, leading to accumulation of DNA errors and eventual tumor cell death.^291^ Several different PARPi (Olaparib, Velaparib, and Rucaparib) have been developed and tested over the past few years with promising results.^292^ In the phase III POLO trial, patients with germline *BRCA1/2* mutations and metastatic PDAC that had not progressed during first‐line platinum‐based chemotherapy were randomly assigned to receive either maintenance Olaparib or placebo.^293^ Median PFS was found to be significantly higher in the Olaparib group compared with the placebo group (7.4 vs. 3.9 mo, HR 0.53, 95% CI 0.35–0.82, *p* = 0.004).^293^ Given the success of the POLO trials, other clinical trials have begun to test the use of Olaparib alone or with in combination with other treatments. A phase II Johns Hopkins study (NCT04753879) is currently recruiting to assess the efficacy of maintenance Olaparib and Pembrolizumab after low dose gemcitabine, nab‐paclitaxel, capecitabine, cisplatin, and irinotecan (GAX‐CI) in patients with untreated metastatic pancreatic cancer.^294^ Similarly, the National Cancer Institute is also conducting a phase II trial (NCT04548752) comparing the use of Olaparib alone versus Olaparib and Pembrolizumab in metastatic germline *BRCA1/2*‐mutated pancreatic cancer.^295^

Other PARPi are currently undergoing similar clinical trials as well. In a phase II trial (NCT03140670), Reiss et al.^296^ found that Rucaparib is safe and effective for patients with metastatic *BRCA1/2*‐ or *PALB2*‐mutated pancreatic cancer with a median PFS of 13.1 months (95% CI 4.4–21.8 months) and median OS of 23.5 months (95% CI 20–27 months). The use of Niraparib and dostarlimab (anti‐PD‐1) on *BRCA1/2* and *PALB2*‐mutated metastatic pancreatic cancer is currently being tested in a phase II trial (NCT04493060).^297^

### 9.2 Targeting somatic mutations

Testing for somatic genetic alterations with available targeted therapy is recommended for patients with LAPC or metastatic PDAC suitable for therapy.^298^ While *KRAS* may be the most common mutation in PDAC, present in 90–95% of tumors, developing an inhibitor targeting *KRAS* mutations have been difficult due to lack of a high affinity binding sites for drugs.^299^, ^300^ In 2018, Janes et al.^301^ found a small molecular compound, ARS‐1620, that was capable of binding to a previously discovered allosteric binding pocket in a specific *KRAS* mutation (KRAS^G12C^) and sequester it in its GDP‐bound inactive form. ARS‐1620 was shown to have antitumor effects via inhibition of phosphor‐ERK, phosphor‐S6, and phospho‐AKT in KRAS^G12C^ preclinical models, leading to the development of other KRAS^G12C^ inhibitors (e.g., Sotosarib, Agadrasib).^301^ And while in 2021, Sotorasib (AMG510) and Adagrasib (MRTX849) received FDA approval and FDA breakthrough therapy designation respectively, the impact of these inhibitors will be limited as KRAS^G12C^ mutation is only present in 1% in PDAC cases.^302^, ^303^, ^304^, ^305^ However, multiple inhibitors targeting other KRAS mutations, pan‐KRAS inhibitors, SHP2 inhibitors, and therapeutic vaccines targeting KRAS are under development and testing.^306^ MRTX1133, a promising, novel KRAS^G12D^ inhibitor, is awaiting clinical trial after having shown preclinical efficacy—eight out of 11 KRAS^G12D^‐mutated cell line xenografts and patient‐derived xenografts showed tumor regression after MRTX1133 treatment.^47^, ^307^ *BI 1701963*, which is currently under phase I trial, is a pan‐KRAS inhibitor that disrupts the binding of KRAS to GEFs SOS1.^308^ Additionally, after promising initial safety and tolerability were reported for SHP2 inhibitor TNO155, multiple clinical trials are being conducted the drug in combination with other therapies.^309^ Table 4 below provides a list of ongoing clinical trials targeting KRAS and its upstream and downstream pathways.

**TABLE 4:** Ongoing randomized clinical trials on therapy targeting KRAS and associated pathways

Trial	Phase	(Target) sample size	Patient type	Treatment regimen	Molecular target	Primary outcome	Status
Chiorean et al.^310^ NCT02451553	Phase I/IB	41	Solid tumors	Afatinib Dimaleate + Capecitabine	EGFR/HER2 tyrosine kinase inhibitor	Safety and toxicity	Results pending
Hoffman et al.^311^ NCT04111458	Phase I	71	Solid tumors	BI 1701963 (+Trametinib)	SOS1/KRAS inhibitor	Dose escalation and confirmation	Active, not recruiting
Ning et al.^312^ NCT05163028	Phase I	42	Solid tumors	HBI‐2376	SHP2 inhibitor	Safety and maximum tolerated dose	Recruiting
KRYSTAL‐1^313^ NCT03785249	Phase I/II	740	Solid tumors with KRAS G12C Mutation	MRTX849 (adagrasib)	KRAS G12C inhibitor	Safety and pharmacokinetics	Recruiting
KRYSTAL‐2^314^ NCT04330664	Phase I/II	86	Advanced solid tumors with KRAS G12C Mutation	MRTX849 + TNO155	KRAS G12C inhibitor SHP2 inhibitor	Safety and pharmacokinetics	Active, not recruiting
Smaglo et al.^315^ NCT03608631	Phase I	28	Metastatic pancreatic cancer with KRAS G12D mutation	Mesenchymal stromal cells‐derived exosomes with KRAS G12D siRNA	KRAS G12D	Dosage, OS, PFS	Recruiting
Hendifar et al.^316^ NCT04390243	Phase II	29	Pancreatic cancer patients with somatic BRAF V600E mutation	Binimetinib + Encorafenib	MEK inhibitor/RAF inhibitor	Efficacy	Recruiting

OS, overall survival; PFS, progression‐free survival.

### 9.3 Microsatellite instability

Microsatellite instability (MSI) represents another subset of PDAC characterized by deficits in the DNA mismatch repair, which can occur as a result of genetic disorders like Lynch syndrome.^317^ Studies have found that compared with microsatellite stable tumors, MSI‐high tumors are more responsive to FOLFIRINOX and comparatively less responsive to 5‐Fu and gemcitabine.^18^, ^318^ Furthermore, it has been reported MSI‐high tumors are more responsive to immune checkpoint inhibitor therapy, and Pembrolizumab (anti‐PD‐1) is now FDA approved for MSI‐high cancers.^319^ In the phase II KEYNOTE‐158 trial, researchers explored the efficacy of Pembrolizumab in noncolorectal MSI‐high or mismatch repair deficient cancers: out of the 22 patients with MSI‐pancreatic cancer, there was an overall response rate of 18.2% (one complete response and three partial responses), suggesting a meaningful response despite it being lower than the overall response rate of all noncolorectal cancers in the study (34.3%).^320^, ^321^

### 9.4 Molecular subtypes of PDAC

Using analysis of primary PDAC RNA expression profiles, several studies have defined different molecular subtypes of PDAC and their associated transcriptional networks.^322^ Several classification methods have been developed, but integration of these methods reveal two basic subtypes of PDAC: classical and basal.^323^, ^324^, ^325^ Classical subtypes show higher expression of epithelial and pancreatic lineage markers and are correlated with a better prognosis; on the other hand, basal subtypes are enriched for cell cycle progression, TGF‐ß signaling, and EMT and are correlated with a poorer prognosis.^326^ Studies have begun exploring the clinical significance of these molecular subtypes in terms of treatment regimen.^326^

To understand the clinical significance of different molecular subtypes, the COMPASS trial recruited patients with metastatic pancreatic cancer prior to first‐line therapy.^327^ Patient tumors were (RNA) sequenced, and tumors were classified into classical (80%) and basal (20%).^327^ Worse prognosis for basal tumors were confirmed with a 60% radiographic progression rate for basal tumors compared with the 15% of classical tumors.^327^ *GATA6* expression in the tumor was identified as a suitable surrogate biomarker for differentiating between molecular subtypes and predict chemosensitivity.^327^ Building on this, the Pancreatic Adenocarcinoma Signature Stratification for Treatment (PASS‐01) trial is currently recruiting and randomizing metastatic PDAC patients to either mFFX or GnP.^328^ This trial will explore the use of molecular profiling, *GATA6* expression, and chemosensitivity signatures to predict response to therapy.

Furthermore, in the ongoing phase III ESPAC‐6 trial (NCT05314998), resected PDAC patients are randomized to either oxaliplatin‐ or gemcitabine‐based adjuvant chemotherapy by either standard clinical criteria (control arm) or transcriptomic treatment specific signature (experimental arm).^329^ The trial seeks to determine whether allocating patients based on transcriptomic stratification is superior to using the standard clinical criteria by comparing DFS and OS. Ultimately, identifying transcriptional subtypes could offer patients more selective chemotherapy treatments, but further understanding on the mechanisms and clinical significance of these subtypes is required.

### 9.5 Targeted therapy in NAT

Overall, there is a lack of research on the use of targeted therapy neoadjuvant style; most research is still currently focused on the efficacy of the targeted therapies as a second‐ or third‐line therapy and/or in conjunction with chemotherapy in an adjuvant manner. However, results of NGS can certainly help determine the NAT regimen. For example, in the case of *BRCA1/2* mutations, FOLFIRINOX and other platinum‐based chemotherapy should be chosen as the NAT regimen if patient performance status allows. Ultimately, there is an urgent need to not only identify possible therapeutic targets and their associated treatments, but to also test their efficacy in both adjuvant and neoadjuvant settings to overcome the immunosuppressive and chemoresistant nature of PDAC.

## 10 CONCLUSION

Despite the poor outlook of PDAC, advancements in surgical techniques, development of combination chemotherapies, and the incorporation of adjuvant therapy have significantly extended OS and DFS. More recently, there has been a shift to delivering chemotherapy in a neoadjuvant fashion with the goal of prolonging OS and DFS. However, the use and assessment of NAT in PDAC have yet to be fully established. Currently, most research support the use of NAT for BRPC and LAPC patients to not only shrink the tumor, but to also select patients who may benefit from surgical resection and increase R0 resection rate. As for patients with resectable pancreatic cancer, current guidelines suggest the use of NAT only in the event of high‐risk features, otherwise upfront surgery is recommended. Given the heterogeneity of patient response to NAT, future research should identify and test reliable and clinically significant biomarkers that can predict patient response. This allows for selection of patients who will benefit from NAT and patients who would not comparatively and should undergo upfront surgery.

Once patients begin NAT, a multifaceted approach including imaging, CA 19‐9, and a histopathologic response should be employed to determine patient response and treatment plan moving forward. Despite FOLFIRINOX being the most commonly used NAT combination chemotherapy, questions regarding the best regimen, dosage, and number of cycles still need to be addressed. Finally, advancements in sequencing technology and a better understanding of tumor biology of have allowed us to enter the era of personalized medicine for the treatment of PDAC. Patients diagnosed with PDAC are recommended to undergo germline testing for cancer predisposing genes to identify more targeted therapies beyond conventional chemotherapy. Most notably, platinum‐based chemotherapy and PARPi should be considered for patients with HDR‐related mutations. However, most targeted therapies are still undergoing clinical trials for adjuvant use, but with promising results. And ultimately, as these therapies are proven to be effective, RCTs should be conducted their use in a neoadjuvant fashion.

## AUTHOR CONTRIBUTIONS

H. K. contributed to the conceptualization, data gathering, drafting, and revising of the manuscript. J. Y. contributed to the conceptualization, data gathering, drafting, and revising of the manuscript. All authors have read and approved the manuscript.

## CONFLICT OF INTEREST STATEMENT

All authors have approved of the manuscript and declare no potential conflicts of interest.

## ETHICS STATEMENT

Not applicable.

## Data Availability

Not applicable.
